# Brain white matter correlates of learning ankle tracking using a wearable device: importance of the superior longitudinal fasciculus II

**DOI:** 10.1186/s12984-022-01042-2

**Published:** 2022-06-27

**Authors:** Chishan Shiao, Pei-Fang Tang, Yu-Chen Wei, Wen-Yih Isaac Tseng, Ta-Te Lin

**Affiliations:** 1 School and Graduate Institute of Physical Therapy, College of Medicine, National Taiwan University, Taipei, Taiwan; 2 Graduate Institute of Brain and Mind Sciences, College of Medicine, National Taiwan University, Taipei, Taiwan; 3 Center for Artificial Intelligence and Robotics, National Taiwan University, Taipei, Taiwan; 4 Neurobiology and Cognitive Science Center, National Taiwan University, Taipei, Taiwan; 5 Department of Physical Medicine and Rehabilitation, National Taiwan University Hospital, Taipei, Taiwan; 6 Institute of Medical Device and Imaging, College of Medicine, National Taiwan University, Taipei, Taiwan; 7 Department of Biomechatronics Engineering, College of Bio-Resources and Agriculture, National Taiwan University, Taipei, Taiwan

**Keywords:** Wearable device, Machine-computer interface, Online visual feedback, Interactive ankle learning, Motor sequence learning, Task-specific, Diffusion-weighted spectrum imaging, Magnetic resonance imaging, Middle-aged, Older adults

## Abstract

**Background:**

Wearable devices have been found effective in training ankle control in patients with neurological diseases. However, the neural mechanisms associated with using wearable devices for ankle training remain largely unexplored. This study aimed to investigate the ankle tracking performance and brain white matter changes associated with ankle tracking learning using a wearable-device system and the behavior–brain structure relationships in middle-aged and older adults.

**Methods:**

Twenty-six middle-aged and older adults (48–75 years) participated in this study. Participants underwent 5-day ankle tracking learning with their non-dominant foot using a custom-built ankle tracking system equipped with a wearable sensor and a sensor-computer interface for real-time visual feedback and data acquisition. Repeated and random sequences of target tracking trajectories were both used for learning and testing. Ankle tracking performance, calculated as the root-mean-squared-error (RMSE) between the target and actual ankle trajectories, and brain diffusion spectrum MR images were acquired at baseline and retention tests. The general fractional anisotropy (GFA) values of eight brain white matter tracts of interest were calculated to indicate their integrity. Two-way (Sex × Time) mixed repeated measures ANOVA procedures were used to investigate Sex and Time effects on RMSE and GFA. Correlations between changes in RMSE and those in GFA were analyzed, controlling for age and sex.

**Results:**

After learning, both male and female participants reduced the RMSE of tracking repeated and random sequences (both *p* < 0.001). Among the eight fiber tracts, the right superior longitudinal fasciculus II (R SLF II) was the only one which showed both increased GFA (*p* = 0.039) after learning and predictive power of reductions in RMSE for random sequence tracking with its changes in GFA [*β* = 0.514, *R*^*2*^ change = 0.259, *p* = 0.008].

**Conclusions:**

Our findings implied that interactive tracking movement learning using wearable sensors may place high demands on the attention, sensory feedback integration, and sensorimotor transformation functions of the brain. Therefore, the SLF II, which is known to perform these brain functions, showed corresponding neural plasticity after such learning, and its plasticity also predicted the behavioral gains. The SLF II appears to be a very important anatomical neural correlate involved in such learning paradigms.

**Supplementary Information:**

The online version contains supplementary material available at 10.1186/s12984-022-01042-2.

## Background

Smooth sequential ankle movements are essential for safe walking and balance, particularly for older adults. Ankle movement learning in older adults and patients with neurological disorders is very common to improve their balance, walking, and mobility [1–3]. Without smooth ankle movements, risks of tripping or slipping may increase, leading to falls and associated medical problems [4, 5].

Various types of sensor-based wearable devices have been developed, applied to ankle movement rehabilitation in patients with neurological disorders, and found to be effective [2, 3, 6]. Researchers have also found multiple benefits of using wearable devices for interactive ankle movement learning, including convenience, cost-effectiveness, provision of real-time feedback, user motivation facilitation, attention enhancement, and availability in the home environment [3, 6]. Moreover, sensor-based ankle movement learning with real-time feedback facilitates more improvement in ankle control than does learning without feedback, suggesting that particular neural mechanisms may be involved in sensor-based learning with feedback to enhance motor learning [2, 7]. Therefore, thorough understanding of the specific neural mechanisms associated with sensor-based ankle movement learning with feedback is important. However, most previous studies have only examined brain activations while performing cyclic ankle movements using functional Magnetic Resonance Imaging (fMRI) [8–15]. The underlying structural and functional neural mechanisms and task-specific brain plasticity associated with sequential ankle movement learning using a sensor-based wearable device and feedback remain little explored. Understanding these neural mechanisms may shed light on how to choose the populations suitable for such learning paradigms.

Motor control studies investigating functional brain activation during self-paced cyclic ankle movements reported brain activation in the bilateral primary motor and sensory cortices, supplementary motor areas, cingulate motor areas, putamen, and cerebellum, with slight right dominance when the tasks were performed with the left ankle [8–13]. Studies that required participants to accurately track a target moving in a sinusoidal wave on a screen with ankle dorsiflexion and plantarflexion movements found additional activation of parietal associative regions as compared to that in simple tasks [14, 15], suggesting that ankle tracking tasks require more sensorimotor integration and attention than do self-paced ankle tasks. However, it remains unknown what brain structures are involved in learning sequential ankle tracking in learning paradigms and the relationship between changes in brain structures and tracking performance after learning.

In previous motor learning studies, researchers have found brain structural correlates of motor learning, but they have primarily focused on upper extremity movements [16, 17]. Bennet et al. and Vien et al. found better integrity of corpus callosal fibers, and the corticospinal tract, hippocampus–dorsolateral prefrontal cortex (DLPFC) tract and caudate–DLPFC tract were associated with better learning of a finger serial reaction time task in both young and older adults [16, 17]. These findings indicated that projection fiber tracts connecting the striatum and DLPFC are important to movement sequence learning.

Although learning smooth sequential ankle movements is important for older adults and patients with neurological disorders to improve balance and walking function, little research has examined the behavioral-brain structure relationship in learning such movements. Therefore, the purpose of this study was to identify the important white matter tracts involved in learning sequential ankle tracking with movements of the non-dominant foot using a wearable device with real-time feedback in healthy middle-aged and older adults using a 5-day motor learning paradigm. We investigated changes in ankle tracking performance of the non-dominant foot and changes in the integrity of eight tracts of interest after learning, and we performed correlation analysis between these changes. The non-dominant foot was selected to better ensure task novelty and observation of behavioral changes after learning [17, 18]. Since the non-dominant foot is not the foot which a person prefers to use when performing skilled movements in daily activities [19], behavioral changes after ankle tracking learning using this foot may be more easily observed. Based on previous evidence supporting their involvement in ankle movement control, we selected eight white matter tracts: four long association fibers connecting parietal association regions with frontal regions, and four projection fibers linking the striatum and frontal regions or linking the cerebral cortex and spinal cord [1, 8–15]. We hypothesized that the integrity of these white matter tracts would increase after sequential ankle tracking learning and that the increases in integrity would be positively correlated with improvement in ankle tracking accuracy after learning. Furthermore, given the known sex differences in biomechanical properties around the ankle [20–22], we also investigated whether sex affected ankle training effects and the central mechanisms associated with ankle tracking learning in this study. We chose middle-aged and older adults as our research participants for two reasons. First, falls and balance problems during daily activities are more prevalent in middle-aged and older adults than in young adults in the healthy population [23–26]. Second, poor ankle control is also very common in patients with stroke, most of whom are middle-aged and older adults [27–29]. According to the National Health Survey in Taiwan, the prevalence of stroke for middle-aged and older adults was 6.5 and 10.4 times, respectively, of that for younger adults [27]. In addition, a report by World Health Organization indicated that stroke prevalence for middle-aged and older adults was 8.7–12.9 times and 61.0–134.3 times, respectively, of that for younger adults in France, Germany, and Italy [29]. Therefore, how middle-aged and older adults learn and control sequential ankle movements is worth investigating and could serve as a good reference for future studies addressing similar research questions in patients with stroke.

## Methods

### Participants

Participants were recruited from the communities of Taipei City and New Taipei City in Taiwan. The inclusion criteria were as follows: (1) age from 45 to 74 years, (2) right footedness determined by scoring > 0 on the Waterloo Footedness Questionnaire–Revised [19], (3) intact cognitive ability as determined by scoring ≥ 28 on the Mini-Mental State Examination [30], (4) good mobility as determined by scoring ≤ 12 s on the Timed Up & Go test [31], and (5) adequate corrected vision sufficient to see clearly a target of 0.5 cm in diameter displayed on a computer screen 0.7 m away. The exclusion criteria were: (1) any neurological disorders or mental illness, (2) musculoskeletal disorders of the lower extremities or past ankle injuries that affected ankle mobility and range of motion, (3) serious cardiovascular diseases or systematic diseases, or (4) any contraindications for MRI, such as metal implants or claustrophobia. All participants underwent screening to assess their eligibility. The screening included demographic information and clinical assessments of footedness, cognition, the Timed Up & Go Test [31], and visual acuity. Before enrollment, all participants signed an informed consent form approved by the Institutional Review Board of National Taiwan University Hospital.

To determine the sample size of this study, we had to estimate the effect size of 5-day ankle tracking learning on reducing tracking errors first. However, such effect size information was not directly available in the literature because most previous studies used a cross-sectional motor control paradigm, but not an ankle tracking learning paradigm, to examine brain activation during the performance of active ankle movements in healthy young adults [8–13]. Nonetheless, a previous functional MRI study reported a similar extent of improvement in tracking accuracy between short-term practice (3 blocks, 30 s/block) of finger and ankle tracking in young adults [15]. Therefore, we calculated the effect size of a 5-day hand tracking learning experiment on tracking errors based on data presented in Meehan et al. [18]. The estimated effect sizes were 0.67 and 0.77 for learning random and repeated sequences, respectively [18]. Consequently, we conservatively estimated our effect size to be 0.6 for healthy adults’ ankle tracking learning of both types of sequence. By setting a power of 0.8 and a two-tailed alpha level of 0.05 to calculate the sample size needed for our study in G*Power 3.1.9.7, we estimated that a sample of 24 was needed.

### Experimental protocol

The flow diagram of this study is presented in Additional file 1. All volunteers were assessed for eligibility first. After screening, all eligible participants underwent baseline tests, which assessed ankle muscle strength and ankle tracking performance, and a brain MRI scan to acquire brain images and measurements of white matter integrity. The muscle strength of the bilateral ankle dorsiflexors was assessed with a hand-held dynamometer (Lafayette Manual Muscle Tester, Model 01163, CA, USA) [32]. A custom-built ankle tracking system was used to examine the ankle tracking performance. Afterwards, the participants undertook a 5-day ankle tracking learning experiment (12 blocks/day), in which the tracking system used for testing was used for sequential ankle tracking learning. At the retention tests, participants completed the same assessments performed at the baseline tests. The time interval between Day 5 and the retention test, called the retention interval [33], was two days. The retention interval was included for testing of the behavioral gains that participants truly had learned, not confounded by practice [18].

### Instrumentation

**Fig. 1:**
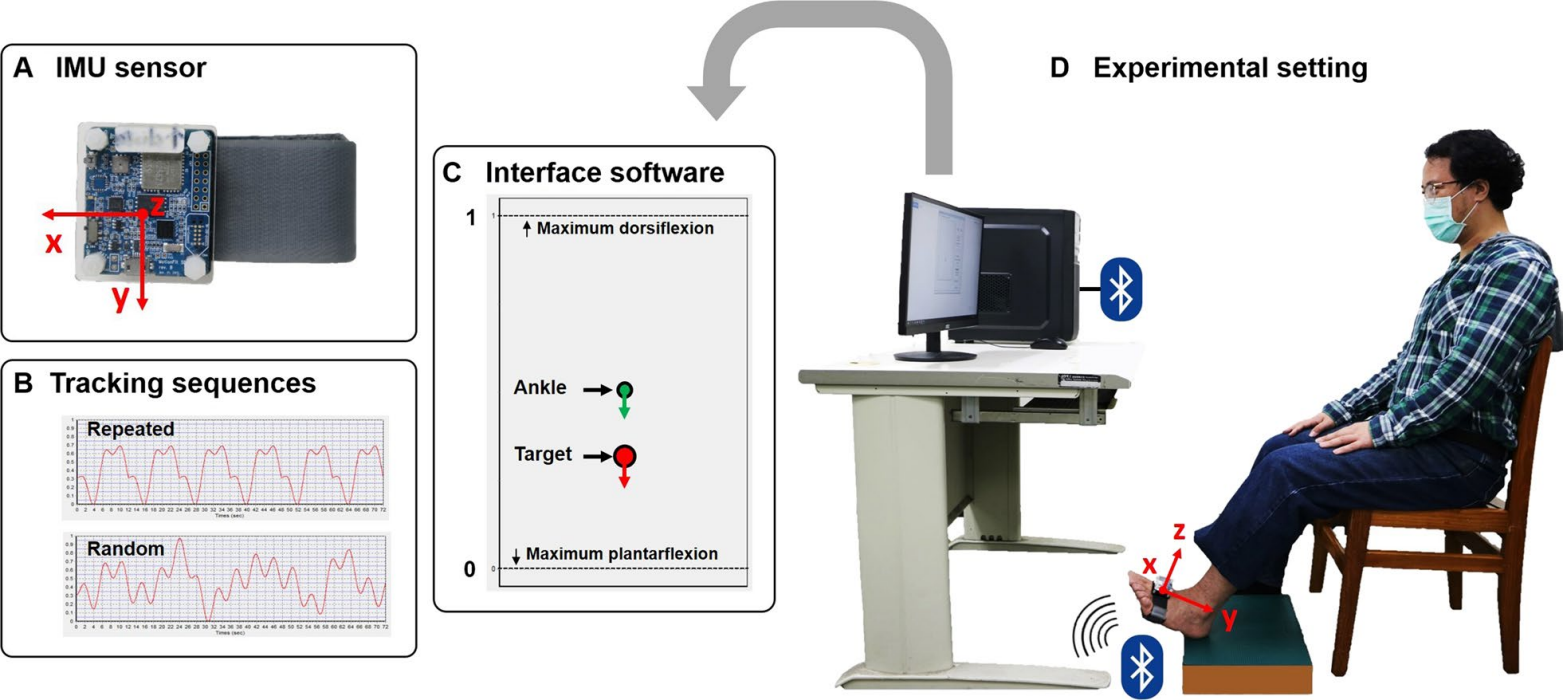
Schematic diagram of the ankle tracking system and experimental setting during testing and learning. **A** The IMU sensor module used in testing and learning. **B** Examples of the 72-s-long repeated (six cycles of the same sequence) and random sequences generated by the interface software of the system and used in the baseline and retention tests. **C** An illustration of the target and ankle locations displaying on the screen to serve as real-time visual feedback to the participant. **D** A diagram of the experimental setting. The participant sat in front of a computer screen in a standard position, with the IMU sensor module worn on the non-dominant foot, and tracked the target cursor as accurately as possible with ankle dorsi- and plantar-flexion of the non-dominant foot. The x-axis of the IMU sensor was aligned to the mediolateral direction of the non-dominant foot, with the positive x, y, and z values on their corresponding axes pointing in the left, posterior, and superior directions, respectively. IMU: inertial measurement unit; MRI: Magnetic Resonance Imaging

The ankle tracking system was composed of a wireless inertial measurement unit (IMU) sensor module (MPU-9150 MotionFit Reference Board, InvenSense®), and custom-built interface software and analysis software. The wireless IMU sensor module consists of an IMU sensor (MPU-9150), an embedded microcontroller, a Bluetooth module for wireless data transmission, and a serial flash memory module on a battery-powered reference board (Fig. 1A). The IMU sensor is a single chip with a 3-degree of freedom (DoF) gyroscope, a 3-DoF accelerometer, and a 3-DoF magnetometer optimized for wearable sensor applications. It can precisely and reliably measure roll, yaw, and pitch angles with less than 0.8° of error.

During testing and learning, the IMU sensor module was fastened on the dorsal aspect of the participant’s non-dominant foot, one centimeter proximal to the metatarsal heads. The x-axis of the IMU sensor was aligned to the mediolateral direction of the non-dominant foot, i.e., parallel to the frontal axis of the ankle, with the positive x, y, and z values on their corresponding axes pointing to the left, posterior, and superior, respectively (Fig. 1D). Therefore, while participants performed ankle dorsiflexion and plantarflexion movements along the frontal axis of the ankle, the IMU sensor could sense rotational movements along its own x-axis. These rotational movements of the IMU were then transformed into an external coordinate frame using a transition matrix to calculate the corresponding ankle dorsiflexion and plantarflexion movements. The average distance from the center of the IMU sensor to the frontal axis of the ankle was used in the transition matrix. In this way, the rotational movements detected by the IMU sensor could be transformed into rotational movements along the frontal axis of the ankle, i.e., the dorsiflexion and plantarflexion movements of the ankle.

The interface software of the ankle tracking system had three functions. First, the software could produce both repeated and random sequences of the trajectory of a target cursor using the polynomial equations described below (Fig. 1B). The experimenters were able to design various experimental protocols by combining blocks of different sequences in various orders. Second, it could display the real-time locations of the target cursor (red circle) and the moving ankle cursor (green circle) on a computer screen (Fig. 1C). The position of the latter was determined by the IMU sensor signal via the coordinate transformation described above. We implemented a calibration procedure by setting the participant’s maximum active ankle dorsi- and plantar-flexion ranges of motion as 1 and 0, respectively, on the screen. In this way, the target cursor would not exceed the maximum ankle range of motion of each participant and the tracking performance of each participant could be calculated relative to his or her own maximum available active ankle range of motion, allowing for between-participant comparisons. The third function of the interface software was to store the location data of the target and ankle cursors at a sampling rate of 62.5 data points per second. The polynomial equations for generating the repeated and random sequences of the cursor trajectory are listed below. The coefficients in the equations were similar to those in previous studies [18, 34].

For the repeated sequence:

1 $$y=c+ {\sum }_{i=1}^{6}{a}_{i}\,\mathrm{sin}\left(2\pi {\omega }_{i}\right)+{b}_{i}\,\mathrm{cos}\left(2\pi {\omega }_{i}\right)$$

where $${c, a}_{i}{, b}_{i}: fixed\, coefficients$$

For a random sequence:

2 $$y=c+ {\sum }_{i=1}^{6}{a}_{i}\,\mathrm{sin}\left(2\pi {\omega }_{i}\right)+{b}_{i}\,\mathrm{cos}\left(2\pi {\omega }_{i}\right)$$

where $${c, a}_{i},{b}_{i}: random\, coefficients$$

The error of the tracking performance in a given trial was calculated by the analysis software of the system as the root-mean-squared-error (RMSE) between the target trajectory and ankle movement trajectory. During learning, this information was given to the participants by the experimenters after each trial. The RMSE score was calculated as:

3 $$RMSE=\sqrt{\frac{1}{n} {\sum }_{i=1}^{n}{({A}_{i}-{T}_{i})}^{2}}$$

where n: number of time points; A_i_: participant’s ankle position at time point *i*; T_i_ : target position at time point *i.*

The RMSE score of every testing trial and every learning block was calculated for later statistical analysis.

### The sequential ankle tracking task

At the baseline and retention tests, participants’ ankle tracking skills were tested using the tracking system. Two trials each of repeated and random sequences were tested. Each trial consisted of six 12-s cycles of repeated sequences or a 72-s-long random sequences, with approximately 36 movement reversals at a movement frequency of 0.083 Hz.

During the 5 days of learning, each participant practiced six 120-s blocks each of repeated and random sequences (i.e., a total of 720 s for each type of sequence) at a movement frequency of 0.083 Hz on each day. In each 120-s block of repeated sequences, participants practiced 10 cycles of the same 12-s sequence. In each random sequence block, the block duration was also 120 s, but the target trajectory was randomly generated by Eq. (2), in which random coefficients were used. The numbers of movement reversals (approximately 60) and difficulty levels were comparable between the repeated and random sequence blocks. The order of repeated and random sequence blocks was randomized on each learning day. Therefore, the design of our learning protocol included variable practice (i.e., using sequences (random and repeated) generated with different coefficients in the polynomial equations) and random practice (i.e., randomized order of repeated and random sequence blocks) to foster effective learning in a short period of time [33, 35]. The determination of the training intensity to be used in this study was referenced to the training intensity used in Meehan et al. and our own pilot study [18]. We chose a slightly higher training intensity than that used in Meehan et al. because we expected foot tracking to be somewhat more difficult than hand tracking [18].

While performing the sequential tracking tasks, each participant wore the IMU sensor on the non-dominant foot and sat in front of a computer screen, which was 0.7 m away from the participant’s chest. Participants’ gaze toward the screen was approximately -30° from horizontal (Fig. 1D). Before each tracking trial, each participant was asked to assume a standard posture of the non-dominant leg with the knee joint at 60 degrees of flexion, the foot supported by a stool, and the heel kept on a fixed point of the stool, which was 0.3 m away from the vertical projection line of the computer screen (Fig. 1D). Then the calibration step was performed to set the participant’s maximum ankle dorsiflexion and plantarflexion ranges as 1 and 0, respectively, on the computer screen. Afterwards, the testing or learning trial began, and all participants were required to perform the tasks with ankle dorsi- and plantar-flexion movements to follow the vertical trajectories of the target as accurately as possible with the initial standard postures being maintained throughout the trial. The experimenters also visually inspected and ensured whether the standard postures were maintained throughout each trial.

### MRI data acquisition and data analysis

A 3-Tesla MR imaging system and a 32-channel head coil (Trio, Siemens, Erlangen, Germany) were used to acquire T1-weighted and diffusion-weighted spectrum imaging (DSI) at National Taiwan University Hospital. High resolution T1-weighted imaging with a magnetization-prepared rapid gradient echo sequence was acquired for the anatomical reference (repetition time/echo time = 2000 ms/2.98 ms, flip angle = 9°, field of view (FOV) = 192 × 256 mm^2^, coronal slice number = 208, and voxel size = 1 × 1 × 1 mm^3^) and for detection of any brain abnormalities. DSI was acquired using a pulsed-gradient spin echo diffusion echo planar imaging sequence (repetition time/echo time = 9600 ms/130 ms, FOV = 200 × 200 mm^2^, matrix size = 80 × 80, slice number = 56, and slice thickness = 2.5 mm). A total of 102 diffusion-encoding directions with the maximum diffusion bmax of 4000 s/mm^2^ were sampled on the grid points in a half sphere of the 3D q-space. The total scan time was approximately 25 min for each participant. During the MRI scan, each participant lay supine with the head fixed in place with cushions and was instructed not to move the head.

We adopted well-validated methods to reconstruct DSI data and calculate tract-based GFA values [36], as in previous studies [37, 38]. First, the quality of the DSI data was checked using a quality assurance pipeline [36]. Then the qualified data were transformed with Fourier transformation to acquire the probability density function [39]. Afterwards, the DSI data were reconstructed and the orientation distribution function (ODF) was computed [40]. The general fractional anisotropy (GFA) value at each voxel was calculated based on the equation of standard deviation of ODF divided by root mean square of ODF. The GFA value ranged from zero, indicating fully isotropic diffusion, to one, indicating diffusion limited to one direction. Greater GFA values indicated greater integrity of white matter tracts.

Subsequently, we used the tract-based automatic analysis (TBAA) method to reconstruct the brain white matter tracts of all participants [36]. The tract atlas based on a standard DSI template, NTU-DSI-122 [41] developed by Chen et al. [36], was used. The T1-weighted and DSI images of all participants were used to create a study-specific template, which was then registered to the standard DSI template. The coordinates of fiber tracts in the standard template were transformed back to individual DSI datasets. The GFA values of each fiber tract were automatically sampled along the coordinates of the tract for each participant.

We analyzed the GFA values of eight fiber tracts of interest in this study. Of these eight tracts, four were association fibers, including the right inferior longitudinal fasciculus (connecting the right temporal pole and the occipital lobe), the right superior longitudinal fasciculus (SLF) I (connecting the right superior frontal gyrus and the right precuneus), the right SLF II (connecting the right triangular part of the inferior frontal gyrus and the right middle occipital gyrus), and the right SLF III (connecting the right opercular part of the inferior frontal gyrus and the right angular gyrus). The other four fiber tracts were projection fibers, including the right corticospinal tract of toe (CST-toe) (connecting the brain stem and the right primary motor cortex of the toe component), the right frontal-striatal tract of the DLPFC (FS-DLPFC) (connecting the right striatum and the right medial and superior frontal gyrus), the right frontal-striatal tract of the precentral gyrus (connecting the right striatum and the right precentral gyrus), and the right thalamic radiation of the DLPFC (TR-DLPFC) (connecting the right thalamus, the right medial and superior frontal gyrus and the supplementary motor area) [36]. The right CST-toe was selected as the control fiber tract because it was anticipated to be responsible primarily for motor production but not motor learning.

### Statistical analysis

Independent t-tests were first used to compare baseline sex differences in demographic and clinical variables, and in the RMSE scores of both sequences and GFA values of 8 fiber tracts. Then 2 (Sex) × 2 (Time) two-way mixed repeated measures ANOVA (RM ANOVA) procedures were performed to evaluate the differences between the baseline and retention tests in the RMSE scores of tracking random and repeated sequences, GFA values of the eight white matter tracts, and strength of ankle dorsiflexors between male and female participants. When a baseline sex difference was found, the RM ANCOVA procedure was used with the baseline value serving as the covariate. Partial correlations were used to evaluate the univariate associations between the changes in GFA values of the eight fiber tracts and the changes in RMSE scores for the two sequences, controlling for age and sex. The changes in GFA values were calculated as the GFA value at retention test minus the GFA value at baseline test, with greater values indicating greater increases in tract integrity after learning. The changes in RMSE scores for each sequence were calculated as the RMSE score at baseline test minus the RMSE score at retention test, and greater changes suggested greater reductions in tracking errors after learning. Furthermore, a stepwise multiple linear regression analysis was performed, controlling for age and sex, to determine whether changes in fiber integrity could predict reductions in tracking errors after learning. Only GFA values of fiber tracts that showed significant findings in the partial correlation analyses were entered into the regression model. The significance level was set at *p* < 0.05 for all statistical analyses. All statistical analyses were conducted in IBM® SPSS® Statistics, version 25 (Wellcome Department of Imaging Neuroscience, London, UK).

## Results

Thirty-two middle-aged and older volunteers passed the screening (see Additional file 1), but 6 of them did not finish the entire study for personal reasons. Therefore, we used the data from the 26 participants who completed the entire study for data analyses (Table 1). All 26 participants (7 males and 19 females) met the inclusion criteria of being right-footed and having intact cognitive ability, good mobility, and adequate visual acuity.

**Table 1:** Demographics and clinical characteristics at baseline

Variables	All (n = 26)	Male (n = 7)	Female (n = 19)	*p*
Demographics				
Age (year)	62.1 ± 8.3 (48.1–74.7)	65.0 ± 8.4 (52.3–74.7)	61.0 ± 8.1 (48.1–73.4)	0.276
Sex (M/F)	7 (27) / 19 (73)	–	–	–
Education (year)	14.5 ± 1.7	14.3 ± 1.8	14.4 ± 1.7	0.861
Weight (kg)	59.1 ± 8.5	69.0 ± 5.0	59.1 ± 9.7	0.804
Height (cm)	159.4 ± 7.6	161.6 ± 7.7	158.5 ± 7.6	0.359
Footedness (L/R)	0/26	0/7	0/19	–
Cognitive function				
MMSE (0–30)	29.3 ± 0.8	28.9 ± 0.9	29.5 ± 0.7	0.076
Motor function				
TUG (s)	8.5 ± 1.4	8.3 ± 1.4	8.6 ± 1.5	0.669
Ankle dorsiflexor strength (kg)				
Dominant ankle	22.5 ± 5.8	27.7 ± 7.4	20.6 ± 3.7	0.045*
Non-dominant ankle	21.9 ± 6.0	27.7 ± 7.1	19.8 ± 3.9	0.025*

Data are presented as mean ± SD (range) or numbers (%)

F: female; L: left; M: male; MMSE: Mini-Mental State Examination; R: right; TUG: Timed Up & Go Test

### Baseline differences

At baseline tests, there were no sex differences for most of the demographic and clinical variables, RMSE scores of repeated and random sequence ankle tracking, or GFA values of fiber tracts (Table 1 and Additional file 2). The only exceptions were that males showed significantly greater ankle dorsiflexor strength (*t* = 2.811, *p* = 0.025) (Table 1) and GFA values of the R FS-precentral fiber tracts (*t* = 3.209, *p* = 0.004) (see Additional file 2). In subsequent two-way analyses for these two variables, RM ANCOVA procedures were used with the baseline values serving as the covariates.

### Changes in behavioral performances and white matter tract integrity after learning

**Fig. 2:**
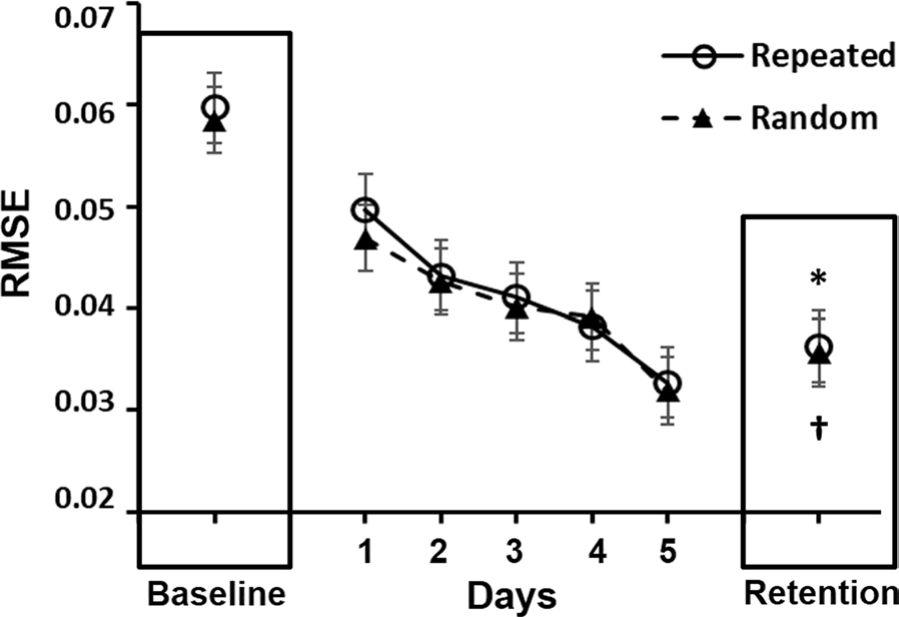
Average tracking performance of all participants at baseline test, during learning, and at retention test. The filled triangles and open circles indicate the average RMSE scores for random and repeated sequences, respectively. * and † indicate significant changes in RMSE scores from baseline to retention tests for repeated and random sequences, respectively. RMSE: root-mean-squared-error

Results of the two-way RM ANCOVA on ankle dorsiflexors showed a significant Time main effect (*p* = 0.008), but no Sex (*p* = 0.111) or Sex × Time interaction effect (*p* = 0.111) (Table 2). Results of the two-way RM ANOVA on RMSE scores showed significant Time effects for both sequences (both *p* < 0.001), but no significant Sex main effects or Sex × Time interactions effects (all *p* > 0.05) (Table 2 and Fig. 2). Both results suggested that sex did not affect the training effects on ankle dorsiflexor strength or RMSE scores. The effect sizes of training effects on RMSE scores were 2.22 and 2.01 for males and 2.22 and 1.86 for females in repeated and random sequence learning, respectively. Partial correlations that further examined whether the increase in the strength of the ankle dorsiflexors was associated with the reductions in RMSE scores, controlled for age and sex, revealed no significant correlations (repeated sequence: *r* = 0.110, *p* = 0.617; random sequence: *r* = 0.145, *p* = 0.510), ruling out the possibility of the contribution of increased ankle strength to improvement in tracking performance.

**Table 2:** Comparison of ankle dorsiflexor strength, RMSE scores, and GFA values between baseline and retention tests

		Baseline test		Retention test		Sex main effect	Time main effect	Time*Sex Interaction
		Male	Female	Male	Female			
Ankle dorsiflexor strength^a^		27.714 ± 7.064	19.805 ± 3.866	26.967 ± 4.770	21.097 ± 2.958	0.111	0.008*	0.111
RMSE								
Repeated sequence		0.058 ± 0.013	0.060 ± 0.014	0.036 ± 0.008	0.036 ± 0.007	0.753	0.000*	0.656
Random sequence		0.056 ± 0.012	0.059 ± 0.017	0.037 ± 0.008	0.035 ± 0.008	0.882	0.000*	0.324
GFA of association fibers								
R ILF		0.409 ± 0.051	0.386 ± 0.038	0.418 ± 0.057	0.387 ± 0.033	0.145	0.182	0.298
R SLF I		0.513 ± 0.037	0.507 ± 0.026	0.513 ± 0.040	0.501 ± 0.023	0.466	0.293	0.359
R SLF II		0.432 ± 0.044	0.434 ± 0.025	0.435 ± 0.042	0.438 ± 0.022	0.874	0.039*	0.756
R SLF III		0.406 ± 0.059	0.401 ± 0.031	0.408 ± 0.054	0.402 ± 0.033	0.747	0.599	0.777
GFA of projection fibers								
R CST-toe		0.596 ± 0.031	0.575 ± 0.027	0.609 ± 0.027	0.578 ± 0.030	0.046*	0.011*	0.120
R FS-DLPFC^a^		0.484 ± 0.037	0.476 ± 0.021	0.478 ± 0.038	0.472 ± 0.021	0.561	0.005*	0.481
R FS-precentral		0.481 ± 0.029	0.446 ± 0.024	0.480 ± 0.042	0.442 ± 0.028	0.831	0.443	0.831
R TR-DLPFC		0.520 ± 0.033	0.509 ± 0.020	0.515 ± 0.034	0.504 ± 0.017	0.272	0.021*	0.919

^a^Baseline value was used as covariate because of baseline sex differences. Data are presented as mean ± SD

^*^p < 0.05

CST: corticospinal tract; DLPFC: dorsolateral prefrontal cortex; FS: frontal-striatum; GFA: general fractional anisotropy; ILF: inferior longitudinal fasciculus; R: right; RMSE: root-mean-squared-error; SLF: superior longitudinal fasciculus; TR, thalamic radiation

Results of the two-way analyses on GFA values of fiber tracts revealed a significant Sex main effect for the R CST-toe (*p* = 0.046), with males presenting a greater GFA value of this fiber tract than females (Table 2). There were significant Time main effects for four fiber tracts, with significant increases in GFA values for the R SLF II (*p* = 0.039) and R CST-toe (*p* = 0.011) and significant decreases in GFA values for the R FS-DLPFC (*p* = 0.005) and R TR-DLPFC (*p* = 0.021) after the ankle tracking learning, compared to baseline. There were no significant Sex x Time interaction effects for all fiber tracts (all *p* > 0.05).

### Correlations between changes in tracking performance and changes in white matter tract integrity after learning

**Fig. 3:**
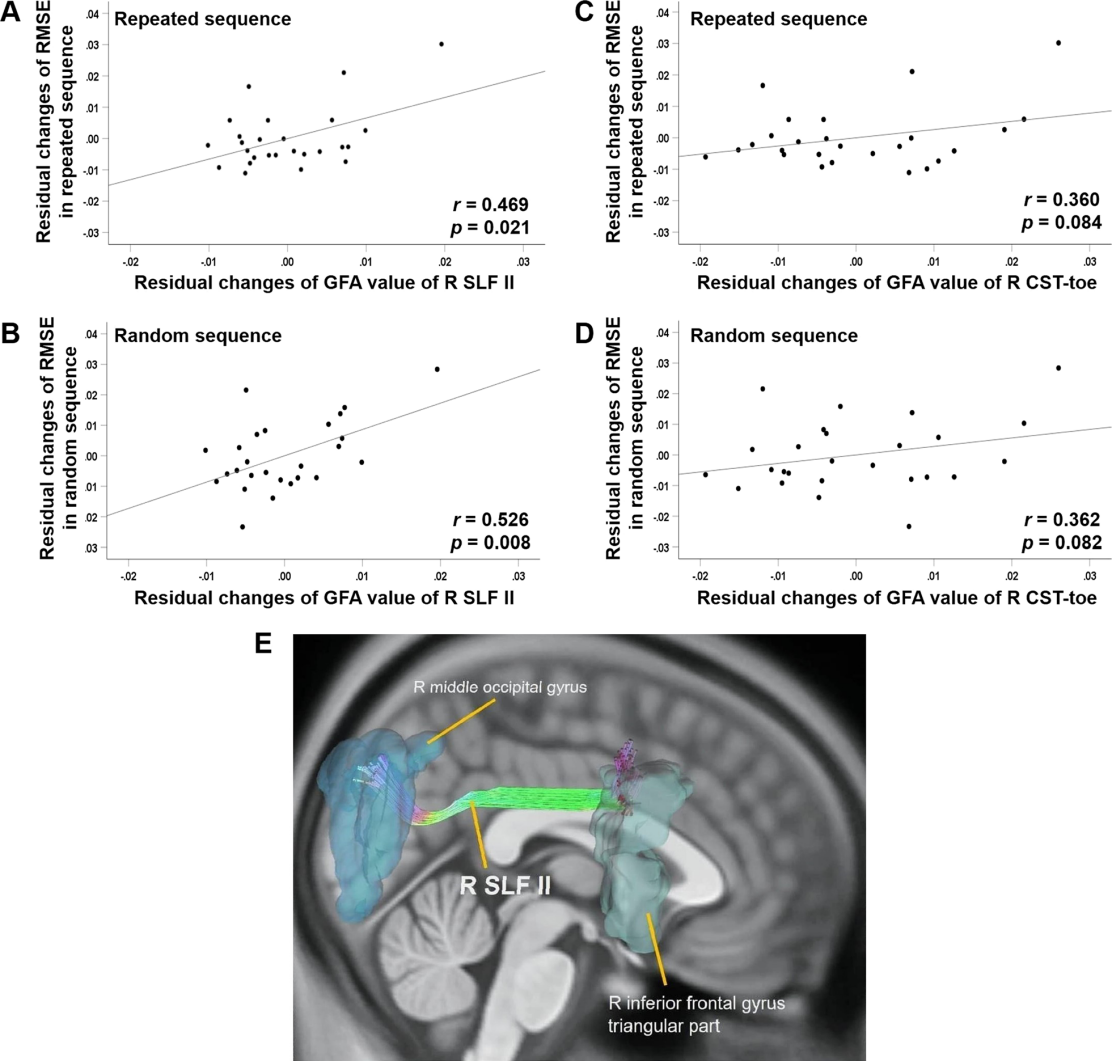
Partial correlations between changes in tracking errors and changes in white matter tract integrity. **A**, **B** Partial correlation plots showed positive correlations between increases in GFA values of the right SLF II and reductions in RMSE scores for **A** repeated (*r* = 0.469, *p* = 0.021) and **B** random (*r* = 0.526, *p* = 0.008) sequences, controlled for age and sex. **C**, **D** Partial correlation plots showed no significant correlations between increases in GFA values of the right CST-toe and reductions in RMSE scores for **C** repeated (*r* = 0.360, *p* = 0.084) and **D** random (*r* = 0.362, *p* = 0.082) sequences, controlled for age and sex. **E** Lateral view of the right SLF II extracted from the tract atlas developed by Chen et al. (2015). CST: corticospinal tract; GFA: generalized fractional anisotropy; R: right; SLF: superior longitudinal fasciculus

The only significant finding from partial correlation analyses was that increases in the GFA values of the R SLF II were significantly correlated with reductions in RMSE scores for both sequences (repeated: *r* = 0.469, *p* = 0.021; random: *r* = 0.526, *p* = 0.008) (Table 3 and Fig. 3A and B). No significant correlations were found between changes in the GFA values of other fiber tracts and reductions in RMSE scores (Table 3 and Fig. 3C and D). Therefore, only the change in GFA values of the right SLF II was entered into the regression model as the potential predictor for RMSE reductions for both sequences. Figure 3E shows the right SLF II and its connected brain regions extracted from the tract atlas created by Chen et al. [36].

**Table 3:** Partial correlations of changes in tract GFA versus changes in RMSE from baseline to retention tests

Fiber name	*r*	*p*
Repeated sequence		
R ILF	0.060	0.782
R SLF I	0.043	0.842
R SLF II	0.469	0.021*
R SLF III	0.311	0.139
R CST-toe	0.360	0.084
R FS-DLPFC	− 0.189	0.376
R FS-precentral	− 0.022	0.918
R TR-DLPFC	0.007	0.974
Random sequence		
R ILF	0.065	0.765
R SLF I	0.071	0.741
R SLF II	0.526	0.008*
R SLF III	0.179	0.402
R CST-toe	0.362	0.082
R FS-DLPFC	− 0.061	0.778
R FS-precentral	− 0.080	0.711
R TR-DLPFC	0.178	0.406

Partial correlation analyses were conducted with age and sex controlled for. Positive correlation values indicate greater increases in GFA values correlate with greater reductions in RMSE scores

CST: corticospinal tract; DLPFC: dorsolateral prefrontal cortex; FS: frontal-striatum; GFA: general fractional anisotropy; ILF: inferior longitudinal fasciculus; R: right; RMSE: root-mean-squared-error; SLF: superior longitudinal fasciculus; TR: thalamic radiation

^*^p < 0.05

The final models of regression analyses revealed that greater increases in GFA values of the right SLF II significantly predicted greater reductions in RMSE scores for random sequence tracking [*β* = 0.514, *R*^*2*^ change = 0.259, *p* = 0.008] and repeated sequence tracking [*β* = 0.468, *R*^*2*^ change = 0.215, *p* = 0.021], after controlling for age and sex (Table 4). However, only the whole model of predicting reductions in RMSE scores of random sequence tracking reached the significance level (*R*^*2*^ = 0.322, *p* = 0.033).

**Table 4:** Final model of multiple linear regression analysis results

	Unstandardized coefficients		Standardized coefficients		R^2^	R2 change	F	*p*
	B	Standard error	*β*	*p*				
Repeated sequence								
Constant	0.009	0.014		0.541				
Age	0.000	0.000	0.175	0.372				
Sex	− 0.002	0.004	− 0.101	0.604				
GFA change of R SLF II	0.651	0.262	0.468	0.021	0.237	0.215	2.272	0.108
Random sequence								
Constant	0.005	0.016		0.758				
Age	0.000	0.000	0.169	0.355				
Sex								
GFA change of R SLF II	0.851	0.293	0.514	0.008	0.322	0.259	3.481	0.033*

The stepwise method was applied. Age and sex were entered as covariates

^*^p < 0.05

GFA, general fractional anisotropy; R, right; SLF, superior longitudinal fasciculus

## Discussion

The present study examined the changes in ankle tracking errors and integrity of eight white matter tracts, as well as the correlations between these changes, after short-term ankle tracking learning with a custom-built sensor-based ankle tracking system in middle-aged and older adults. We found that participants demonstrated significant reductions in ankle tracking errors in tracking repeated and random sequences and significant increases in the integrity of the right SLF II after learning. Moreover, among the eight fiber tracts, the right SLF II was the only fiber tract which showed positive correlations of its increases in integrity with reductions in ankle tracking errors for both sequences. In addition, results of the regression analysis showed that changes in the integrity of the right SLF II predicted the reductions in tracking errors significantly for the random sequence condition (*p* = 0.008) and the repeated sequence condition (*p* = 0.021). Our findings of the neural plasticity of the SLF II associated with ankle tracking learning and its predictive power of behavioral gains suggested the importance of the SLF II in attention, multisensory integration, and sensorimotor transformation processes required in learning ankle tracking using wearable sensors with feedback. To our knowledge, this study was the first one to shed light on the structural neural plasticity correlated to short-term ankle tracking learning using an interactive wearable sensor system.

Two possible reasons may account for the behavioral gains after such a short-term (5 days) learning program. First, the key functional features of our custom-built sensor-based ankle tracking system may have helped to facilitate short-term learning. The interface software of our system provided real-time graphical feedback on the position of the moving ankle relative to the target (i.e., knowledge of performance). In addition, the experimenter gave participants delayed numerical feedback on the RMSE score (i.e., knowledge of results) after each block during skill acquisition [42–45]. Real-time knowledge of performance information may help with the multisensory integration and sensorimotor transformation processes required in learning a motor task demanding high accuracy and keep the participant’s attention on key features of the movement sequences to be learned [46, 47]. The information providing knowledge of results could help the participants develop an internal reference of movement sequences and increase their motivation [16, 42–45].

Second, the learning effects may also be attributed to our use of an effective learning protocol which adhered to motor learning principles, including a sufficient amount of practice and the use of variable and random practice designs. In this study, the training intensity (1440 s/day) was slightly higher than that used in a prior 5-day hand tracking task study (1000 s/day), which already showed significant learning effects on RMSE reductions in healthy adults and patients with stroke at the retention tests [18]. Furthermore, the total amount of practice was 300 sequences for each participant in this study, which was comparable to amounts (168–3600 sequences) reported in previous movement sequence learning studies [16, 18, 48].

The combined use of variable and random practice schedules may likely enhance learning. Variable practice refers to a practice schedule in which different values of the same parameters are used in the same learning task during practice, helping learners both to establish the relationships between internal parameter values and environmental outcomes, the so-called motor schema, and to develop a generalized motor program of movements of the same class [33]. Consequently, the learners could perform better in the retention [49, 50] and transfer tests [51]. In our study, half of the practice was done with random sequences and the other half was done with repeated sequences. We expected that the incorporation of both types of sequences could enhance participants’ learning of the relationships between ankle movements and tracking errors. Random practice refers to the use of a fully random order of task variations [33]. Applications of random practice schedules have also been shown to lead to more effective motor learning than blocked practice, in which no task variations were given within blocks [52–55]. Two hypotheses have been proposed to explain the superior learning effects of random practice. The “elaborative processing hypothesis” suggests that learning different tasks randomly promotes more comparisons among the actions required to complete the tasks, and thus facilitates learning [54, 55]. The “forgetting and reconstruction hypothesis” suggests that random practice promotes learners to forget previous motor plans and reconstruct another plan for the upcoming task, forcing learners into more elaborative conceptual processing of the tasks [52, 53]. As a whole, our combined application of variable and random practice designs and the interactive sensor-based system to provide feedback effectively fostered participants’ learning of novel sequential ankle tracking tasks in a short period of time, even for middle-aged and older adults.

Other important novel findings of our study were the significant increase in the integrity of the right SLF II after 5 days of learning and the predictive power of this increase in integrity for reductions in tracking errors. These findings provide the first evidence in support of the task-specific brain white matter structural plasticity associated with learning ankle tracking tasks using a sensor-based wearable system. Previous studies have also reported associated brain white matter plasticity after specific upper extremity motor skill learning, but without the use of wearable devices to provide real-time feedback. Scholz et al. found that, in young adults, the integrity of fiber tracts in the region of interest at the right posterior intraparietal sulcus, brain regions related to attention and visuomotor transformation, significantly increased after 6 weeks of learning juggling skills [56]. Task-specific brain white matter plasticity was also observed in a finger tapping study, in which young adults learned to produce finger tapping sequences synchronized with visual stimuli in precise timing for 5 days [57]. It was found that after the learning, the FA value of white matter underlying the bilateral sensorimotor cortices was correlated to final synchronization performance [57]. The findings of these two studies, together with ours, suggested that fiber tracts passing the parietal regions may play an important role in learning novel visuomotor tasks with the upper and lower extremities. Our study further extended the findings to motor skill learning in middle-aged and older adults using tract-based analysis of white matter tract integrity.

We speculated that the learning-specific plasticity of the SLF II was due to the fact that ankle tracking learning using a wearable sensor with feedback highly depended on attention, sensory integration, sensorimotor transformation, and action programming processes, all of which are closely related to the key functions of the SLF II [58–60]. First of all, learning a tracking task requires both top-down goal-directed attention to detect the target cursor and self-movement cursor positions and bottom-up stimulus-driven attention to detect ongoing errors of self-movements. The fronto-parietal network serves these dual-attention functions [61, 62]. The dorsal fronto-parietal network, including the intraparietal cortex and superior frontal cortex, is involved in goal-directed selection of stimuli. The ventral part of the network, including the temporoparietal cortex and inferior frontal cortex, is involved in stimulus-driven attention to salient stimuli [61, 62]. The SLF II traverses through the fronto-parietal network; thus, it could provide an important pathway for both top-down and bottom-up spatial attention processes and could assist participants to concentrate on the tracking task. Further evidence for the role of the SLF II in attention can be observed in studies showing that the SLF in the non-dominant hemisphere is especially involved in visuospatial awareness [62, 63]. In patients who had received glioma dissection, Nakajima et al. found that patients with lesions involving the right superior and middle frontal gyrus, which are on the course of the right SLF I and II, showed severe visuospatial cognitive deficits even 3 months after the surgeries [63], suggesting the important role of the right SLF II in visuospatial awareness.

During ankle tracking, the perceived tracking error information needs to be further processed by sensory integration in the parietal association area to integrate inherent ankle joint proprioception with augmented visual feedback of the tracking errors. Sensorimotor integration processes are then needed to pass the integrated sensory information on to the premotor cortices to prepare and formulate programs for correcting ongoing ankle tracking errors. The SLF II traverses the middle occipital gyrus, inferior parietal lobe, intraparietal sulcus, angular gyrus, and posterior regions of the superior and middle frontal gyri [36, 64, 65] and thus plays an important role in sensory information and transferring information between the parietal/occipital and frontal regions [66]. Evidence for the sensory integration and sensorimotor transformation functions of the SLF II has been observed in previous research on grasping and reaching [60, 67]. Buch et al. examined the correlation between brain white matter integrity at baseline and the success rate of controlling a cursor movement through adjusting hand grasping power after 4 weeks of learning the task in patients with stroke [67]. Participants were required to control a cursor movement displayed on a screen to contact a target by adjusting the power of the hand grasping an orthosis designed for the task. It was found that the FA value of the SLF II at baseline was positively correlated with the success rate of cursor control after learning [67]. Thus, the higher FA value of the SLF II at baseline may have enhanced the sensory integration of hand proprioceptive input and visual feedback information and facilitated the learning of the task [67]. In a visually-guided reaching study, young participants were required to reach both undisplaced static targets and unexpected displaced targets [60]. The reaching task was performed before and after a virtual circuit disruption created by applying 1 Hz repetitive transcranial magnetic stimulation (rTMS) at the intraparietal sulcus, a region known to be related to visuomotor transformation for online control of reaching. It was found that after the rTMS, people with higher FA values of the SLF II had lower reductions in reaching accuracy [60]. Results of both studies suggested that greater integrity of the SLF II was related to better sensory integration, sensorimotor transformation, and action programming (or reprogramming) in visuomotor tasks [60, 67].

Furthermore, functional MRI (fMRI) studies using visually-guided tracking tasks have found significant activations in the frontal-parietal regions to which the SLF II is connected, supporting the needs for processing of attention, sensory integration, and sensorimotor transformation while performing these tracking tasks [14, 15]. LaPointe et al. investigated the brain regions associated with visually-guided finger and ankle tracking tasks in young adults using fMRI [15]. They investigated the brain activations in the primary motor cortex, primary somatosensory cortex, supplementary motor area, and premotor cortex during tracking to study the brain organization needed to control continuous accurate tracking movements in visuomotor tasks. In both finger and ankle tracking tasks, the bilateral superior parietal lobes were highly activated [15]. Compared to finger tracking, ankle tracking was associated with greater activation in bilateral supplementary motor areas [15]. These results supported the role of the fronto-parietal network in tracking tasks. Also using fMRI, Cunningham et al. observed brain activations associated with finger, elbow, and ankle tracking in healthy young adults [14]. Participants were required to track complex movements of a target using these joints, separately. Brain regions responsible for motor planning and execution (primary motor cortex, supplementary motor area), sensory processing (primary sensory cortex), and visuospatial attention (superior and inferior parietal lobe) functions were found to be activated in all three types of tracking tasks [14]. These results suggested that the fronto-parietal regions were important in the execution of complex sequential movements under visual guidance that require intense ongoing concentration [14]. To further test the importance of the SLF II in learning tracking tasks with wearable sensors, future studies may recruit patients with intact or lesioned SLF II and compare their improvement after learning.

While we found increased integrity of the right SLF II and CST-toe, we also found decreased integrity of the right TR-DLPFC and FS-DLPFC fiber tracts. One possible explanation for the decreased integrity of the two fiber tracts connecting to the DLPFC may be that the ankle tracking task did not place a heavy demand on the functions of the DLPFC. The primary functions of the DLPFC are known to be decision making and motor planning, especially for self or internally initiated movements such as serial reaction time tasks [16, 18, 68]. Previous motor learning studies employing serial reaction time tasks indeed found increases in the integrity of the hippocampus-DLPFC and caudate-DLPFC tracts after learning, suggesting the internally generated nature of serial reaction time tasks [16]. In contrast, our ankle tracking task was more of an externally triggered task. Therefore, we did not find increased integrity of these DLPFC-related fiber tracts.

Although we also found increased muscle strength of the ankle dorsiflexors in the non-dominant foot as well as increased integrity of the R CST-toe fiber tract, these increases were not related to reductions in tracking errors after learning. Therefore, the participants’ reductions in tracking errors reflected that they indeed had improved the tracking skills; the reductions were not due to increased ankle muscle strength or increased integrity of fiber tracks related to ankle strength. These findings further supported our speculation that the ankle tracking learning task heavily depended on white matter tracts involved in complex sensorimotor processes, rather than those involved in motor production alone.

### Limitations and clinical applications

There were three limitations in the current study. First, we did not use a device to constrain participants’ ankle movements in the coronal plane during testing and learning because we wanted the participants to perform their ankle movements naturally so as to better simulate ankle movements in daily activities. In this unconstrained condition, participants were able to use compensatory ankle movements in the coronal plane to move the sensor. Future research using similar sensor-based training programs will need to incorporate alarm or feedback mechanisms into the sensor system to better ensure the quality of the participant’s movements in the desired plane. Second, biomechanical characteristics of the ankle, such as ligamentous laxity and tendon stiffness, may have an impact on the learning effects and thus need to be taken into consideration in future studies. Third, because we did not have a control condition in which wearable sensors were not used, no definitive conclusions could be drawn on whether the plasticity of the SLF II was due to the tracking task per se or the use of the wearable sensor. Future tracking studies including a control condition without using wearable sensors are needed to answer this question.

The significant positive training effects on behavioral accuracy of sequential ankle tracking and corresponding brain white matter integrity found in our study provide strong support for the applications of using sensor-based ankle tracking systems in the clinics, particularly for patients with neurological disorders, such as patients with stroke, spinal cord injury, or traumatic brain injury, who often present ankle control problems [69–76]. Training to improve ankle control of these patients is challenging in rehabilitation. Previous studies using sensor-based systems on ankle rehabilitation for these patients have mainly focused on discrete or cyclic ankle movement training or strength and proprioception training and the training period needed to last for at least 4 weeks to observe significant functional improvements [2, 3, 48, 77]. Our findings suggest that when applying sensory-based systems to train ankle control in these patients, using more complex sequential ankle movements, pairing with variable and random practice motor learning paradigms, may foster learning and the corresponding brain plasticity in a shorter period of time. Future clinical trials to test the effects of the training protocols and sensor-based system used in this study in patients with neurological disorders are warranted.

## Conclusions

Our study revealed that short-term learning of ankle tracking skills using a wearable sensor with feedback not only significantly improved ankle tracking accuracy, but also induced learning-dependent brain white matter plasticity of the SLF II in middle-aged and older adults. Furthermore, the increase in the integrity of the SLF II positively predicted the amount of the reduction of tracking errors after learning. These findings suggest that, when a sensor-based ankle tracking system providing real-time visual feedback is used to train visuomotor skills, participant’s plasticity of the SLF II may be crucial in determining the behavior gains because the SLF II provides the functions of attention, sensory integration, and sensorimotor transformation, which are heavily required in such learning paradigms.

## Supplementary Information

**Additional file 1**. Flow diagram of the study.

**Additional file 2**. Sex differences in RMSE scores and GFA values at baseline test.

## Data Availability

The datasets generated and/or analyzed during this study are available from the corresponding author on reasonable request. Requests to access the datasets should be directed to pftang@ntu.edu.tw.

## Abbreviations

CST: Corticospinal tract
DLPFC: Dorsolateral prefrontal cortex
DoF: Degree of freedom
DSI: Diffusion-weighted spectrum imaging
fMRI: Functional Magnetic Resonance Imaging
FOV: Field of view
FS: Frontal-striatal
GFA: General fractional anisotropy
IMU: Inertial measurement unit
ODF: Orientation distribution function
RMSE: Root-mean-squared-error
SLF: Superior longitudinal fasciculus
TBAA: Tract-based automatic analysis
TE: Echo time
TR: Thalamic radiation
