# Supplementary figures and images for: Brain white matter correlates of learning ankle tracking using a wearable device: importance of the superior longitudinal fasciculus II
Source: J Neuroeng Rehabil. 2022 Jun 27;19:64. doi: 10.1186/s12984-022-01042-2 (PMC9237986; doi:10.1186/s12984-022-01042-2)

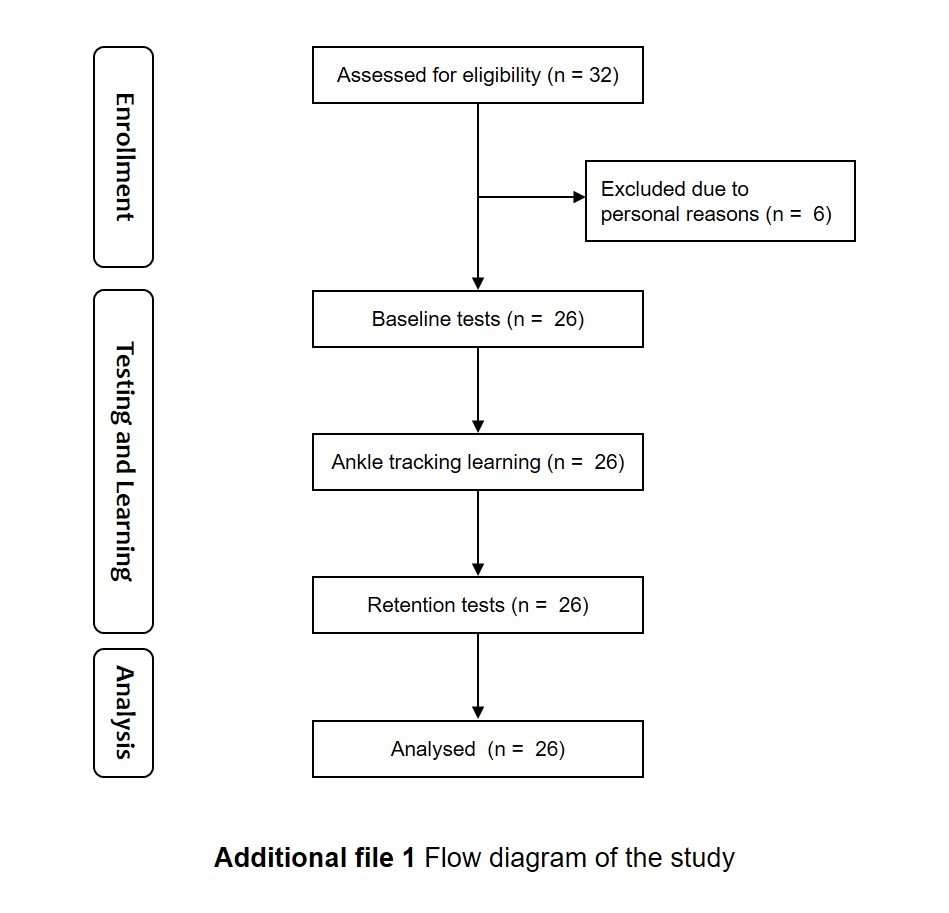

Supplement: Supplementary file 1 — Additional file 1. Flow diagram of the study. [file 12984_2022_1042_MOESM1_ESM.jpg]
