# Supplementary material for: Brain white matter correlates of learning ankle tracking using a wearable device: importance of the superior longitudinal fasciculus II
Source: J Neuroeng Rehabil. 2022 Jun 27;19:64. doi: 10.1186/s12984-022-01042-2 (PMC9237986; doi:10.1186/s12984-022-01042-2)
Supplement: Supplementary file 2 — Additional file 2. Sex differences in RMSE scores and GFA values at baseline test. [file 12984_2022_1042_MOESM2_ESM.docx]

**Additional file 2** Sex differences in RMSE scores and GFA values at baseline test

|  |  | **Male** | **Female** | ***t*** | **95% CI** | | ***p*** | |
| --- | --- | --- | --- | --- | --- | --- | --- | --- |
|  | |  |  |  | **Lower** | **Upper** |  |  |
| **RMSE** | |  |  |  |  |  |  |  |
|  | Repeated sequence | 0.058 ± 0.013 | 0.060 ± 0.014 | -0.391 | -0.015 | 0.010 |  | 0.699 |
|  | Random sequence | 0.056 ± 0.012 | 0.059 ± 0.017 | -0.472 | -0.018 | 0.011 |  | 0.641 |
| **GFA of association fibers** | |  |  |  |  |  |  |  |
|  | R ILF | 0.409 ± 0.051 | 0.386 ± 0.038 | 1.237 | -0.015 | 0.060 |  | 0.228 |
|  | R SLF I | 0.513 ± 0.037 | 0.507 ± 0.026 | 0.510 | -0.020 | 0.033 |  | 0.615 |
|  | R SLF II | 0.432 ± 0.044 | 0.434 ± 0.025 | -0.117 | -0.030 | 0.027 |  | 0.908 |
|  | R SLF III | 0.406 ± 0.059 | 0.401 ± 0.031 | 0.206 | -0.051 | 0.060 |  | 0.842 |
| **GFA of projection fibers** | |  |  |  |  |  |  |  |
|  | R CST-toe | 0.596 ± 0.031 | 0.575 ± 0.027 | 1.717 | -0.004 | 0.047 |  | 0.099 |
|  | R FS-DLPFC | 0.484 ± 0.037 | 0.476 ± 0.021 | 0.689 | -0.016 | 0.032 |  | 0.498 |
|  | R FS-precentral | 0.481 ± 0.029 | 0.446 ± 0.024 | 3.209 | 0.013 | 0.058 |  | 0.004* |
|  | R TR-DLPFC | 0.520 ± 0.033 | 0.509 ± 0.020 | 1.087 | -0.010 | 0.034 |  | 0.288 |

Data are presented as mean ± SD.

**p*< 0.05.

Abbreviations: CST, corticospinal tract; DLPFC, dorsolateral prefrontal cortex; FS, frontal-striatum; GFA, general fractional anisotropy; ILF, inferior longitudinal fasciculus; R, right; RMSE, root-mean-squared-error; SLF, superior longitudinal fasciculus; TR, thalamic radiation.
